# Multicentre Evaluation of an AI‐Assisted Urine Test for Clinically Significant Prostate Cancer in Men Undergoing Initial Biopsy

**DOI:** 10.1002/jev2.70233

**Published:** 2026-03-25

**Authors:** Shaoqin Jiang, Chunguang Yang, Zhangcheng Huang, Zebang Guo, Feiting Lu, Xinwen Nian, Zhenlin Chen, Pengwei Luo, Jiawei Jiang, Xu Gao, Mengqiang Li, Fei Liu

**Affiliations:** ^1^ Department of Urology Fujian Union Hospital, Fujian Medical University Fuzhou Fujian China; ^2^ Department of Urology Tongji Hospital, Tongji Medical College, Huazhong University of Science and Technology Wuhan Hubei China; ^3^ Department of Urology The Second People's Hospital, Fujian University of Traditional Chinese Medicine Fuzhou Fujian China; ^4^ Shenzhen Huixin Lifetechnologies Co., Ltd. Longhua, Shenzhen Guangdong China; ^5^ Department of Urology Changhai Hospital Shanghai China; ^6^ Department of Medicine Brigham and Women's Hospital, Harvard Medical School Boston Massachusetts USA

## Abstract

The Extracellular Vesicles Gene‐based Prostate Score (EGPS), powered by DeepSeek, is an artificial intelligence (AI) diagnostic tool that enhances the detection of clinically significant prostate cancer (csPCa) using urinary EV‐derived gene expression, without requiring digital rectal examination (DRE). To address overdiagnosis resulting from the limited specificity of prostate‐specific antigen (PSA) and reduce unnecessary biopsies, this study evaluated the clinical utility and generalizability of EGPS in men undergoing initial biopsy with PSA levels ranging from 0 to 15 ng/mL.

A total of 645 patients were retrospectively enrolled: 586 from three centres were divided into training (70%) and internal validation (30%) cohorts, and 59 from two centres served as the external validation cohort. EVs were isolated using the EXODUS platform, and gene expression was measured by RT‐qPCR. Ten machine learning algorithms were evaluated for constructing the EGPS model with selected genes. Diagnostic efficacy was assessed by ROC analysis, DeLong tests, and decision curve analysis. An AI diagnostic system using DeepSeek was also developed.

The EGPS model, incorporating AMACR, HOXB13, and PSGR, achieved AUCs of 0.838, 0.825, and 0.811 in the training, internal validation, and external validation cohorts, respectively, outperforming PSA. At a cut‐off value of 0.22, the model demonstrated sensitivity above 95%, with a missed diagnosis rate of 3.81% in the training cohort and 0% in the validation cohorts. The model reduced unnecessary biopsies by 79 (23.37%), 27 (18.62%) and 9 (15.25%) cases across the three cohorts, thereby lowering biopsy‐related risks. A DeepSeek‐powered AI diagnostic system integrating EGPS was developed to support csPCa diagnosis and minimize unnecessary biopsies.

EGPS, derived from multicentre Chinese cohorts, enables accurate, DRE‐free, non‐invasive prediction of csPCa in men with PSA levels of 0–15 ng/mL. When integrated into an AI system, EGPS supports early screening and personalized clinical decision‐making by reducing unnecessary biopsies.

## 1 Introduction

Prostate cancer (PCa) is one of the most prevalent genitourinary malignancies in older men, accounting for up to 30% of newly diagnosed male cancers in the United States by 2025 (Siegel et al., 2025). Serum prostate‐specific antigen (PSA) remains the primary screening method for PCa (Qaseem et al., 2013). However, elevated PSA can occur in benign conditions, such as prostatitis and benign prostatic hyperplasia (BPH), thereby reducing its specificity for diagnosing PCa (Welch & Albertsen, 2020; Duffy, 2020). Currently, prostate biopsy remains the gold standard for confirming PCa. Nevertheless, it is invasive and is associated with complications, including bleeding, infection, pain, as well as additional financial burdens for patients (Fenton et al., 2018). Only <30% of biopsies are positive when PSA is 10–20 ng/mL, with even lower rates in the grey zone (4–10 ng/mL) (Li & Na, 2008; Xiong et al., 2016). Moreover, 42.3% of biopsy‐diagnosed PCa cases are non‐clinically significant prostate cancer (non‐csPCa) (Herget et al., 2016), which typically progresses slowly and often only requires active surveillance due to quality‐of‐life considerations (Litwin & Tan, 2017). Current evidence indicates that PSA‐guided biopsies result in unnecessary biopsies for approximately 60% of patients, substantially increasing patient burden and procedure‐related risks (Na et al., 2017; Bjurlin et al., 2014). Therefore, more accurate screening tools are urgently needed to improve specificity and reduce unnecessary biopsies.

Urine, which contains urogenital secretions and cellular debris, offers a non‐invasive alternative to blood‐based PSA tests for PCa biomarker research (Jain et al., 2023). Models, such as Progensa PCA3, the Mi‐Prostate Score (MiPS), and Select MDx, use urine RNA following a digital rectal examination (DRE) to diagnose clinically significant prostate cancer (csPCa) (Hologic 2024; Tomlins et al. 2016; Tosoian et al. 2021; Haese et al. 2019). However, the DRE may cause discomfort, reducing patient compliance with this procedure (Romero et al. 2008). Urinary extracellular vesicles (EVs) can carry highly conserved miRNAs and mRNAs in lipid‐bound vesicles (Maia et al. 2018). Originating from endosomes or the plasma membrane, EVs play a critical role in intercellular communication, and accumulating evidence indicates that urinary EVs can serve as a practical, non‐invasive diagnostic tool (Jain et al. 2023; Drake et al. 2009; Zhu et al. 2021). Notably, RNA inside EVs is more stable than RNA from whole urine or sediment (Merchant et al. 2017). Thus, RNA from EVs in non‐DRE urine can be used to construct predictive models. An Exosome‐based Prostate Index (EPI) model using non‐DRE urine‐derived EVs shows promise in predicting csPCa while reducing unnecessary biopsies (Donovan et al. 2015; McKiernan et al. 2016). However, this model was developed based on Western populations, thus limiting its applicability to Asian populations (Kong et al. 2020). Hence, there is an urgent need to develop a csPCa diagnostic model based on non‐DRE urine EVs tailored specifically for Chinese populations.

**FIGURE 1:**
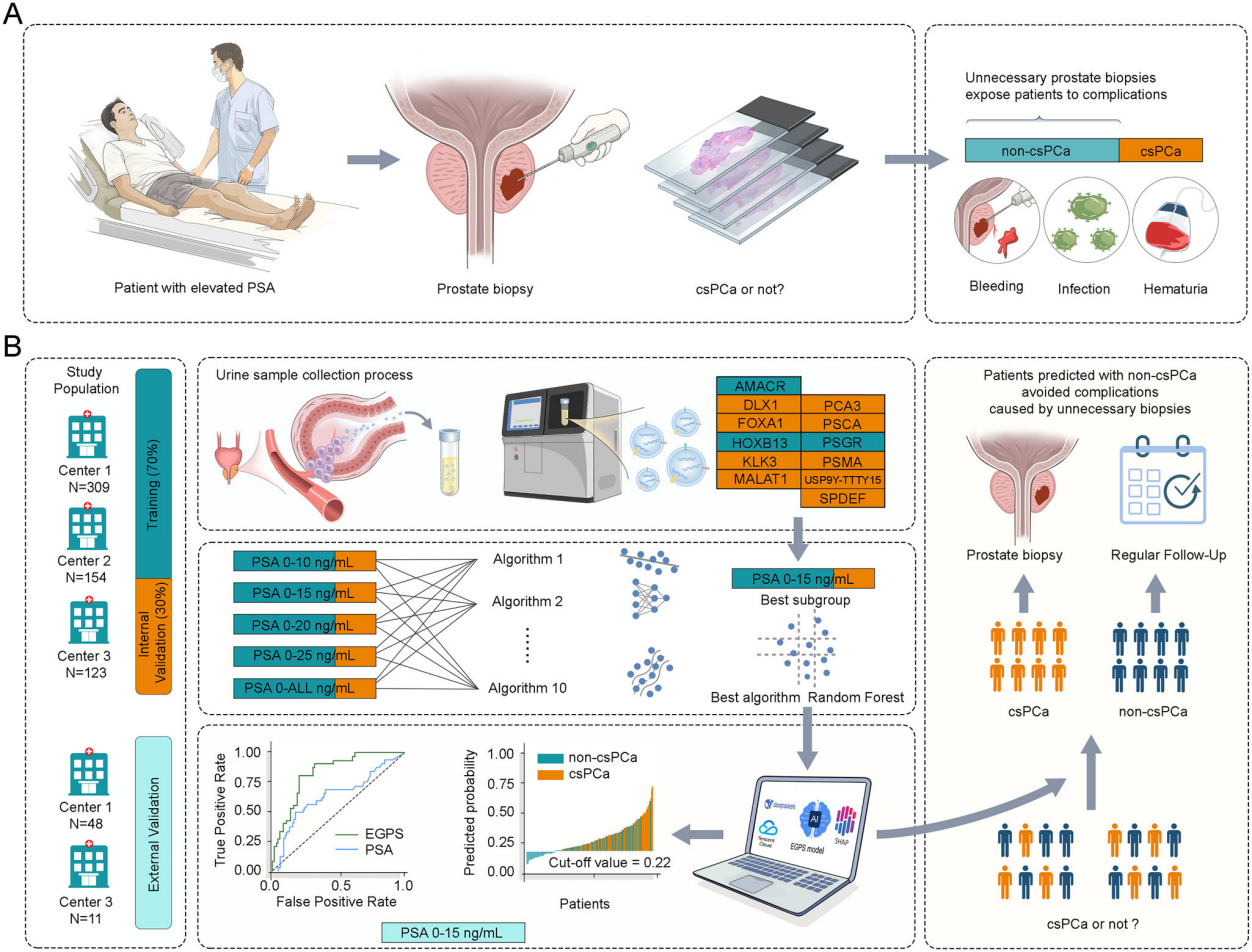
Development workflow and clinical application of the EGPS model for non‐invasive prediction of clinically significant prostate cancer (csPCa). (A) Schematic representation of clinical challenges associated with traditional PSA‐guided prostate biopsy. Patients with elevated PSA levels undergo prostate biopsy to assess the presence of csPCa. However, due to the limited specificity of PSA, many patients with the non‐csPCa are exposed to unnecessary biopsies, leading to potential complications such as bleeding, infection, and haematuria. (B) Overview of the development and implementation of the EGPS diagnostic model. From May 2022 to March 2025, urine samples were collected from patients scheduled for initial biopsy across three Chinese medical centres. Samples were processed using the EXODUS platform to isolate EVs, from which gene expression of candidate prostate cancer biomarkers (e.g., AMACR, HOXB13, PSGR) was quantified via RT‐qPCR. Multiple machine learning algorithms were trained and evaluated across five PSA subgroups to determine the optimal diagnostic window, with the 0–15 ng/mL PSA subgroup and random forest algorithm yielding the best performance. The final EGPS model demonstrated superior accuracy compared to PSA, as shown by ROC and waterfall plots. An intelligent online diagnostic system integrating SHAP explainability and DeepSeek‐based AI recommendations was developed to facilitate personalized, real‐time clinical decision‐making. Patients predicted to have csPCa were referred for biopsy, while those classified as non‐csPCa were advised to undergo routine follow‐up, thereby avoiding unnecessary invasive procedures. EGPS, Extracellular vesicles Gene‐based Prostate Score (including AMACR, HOXB13, and PSGR).

In our previous research, we successfully isolated and detected urinary EVs using the fully automated ultrafast‐isolation system (EXODUS) (Chen et al. 2021) and established a related gene‐detection method (Jiang et al. 2024). Based on these advanced methods, our study conducted a retrospective analysis across three Chinese medical centres on patients undergoing initial prostate biopsy. As illustrated in Figure 1, non‐DRE urine samples were collected to extract EVs containing the AMACR, HOXB13, and PSGR genes, which were subsequently utilized to construct the Extracellular vesicles Gene‐based Prostate Score (EGPS) model. This model demonstrated significantly enhanced diagnostic efficacy for csPCa. We further developed a DeepSeek‐powered artificial intelligence (AI) diagnostic system to support data interpretation and model refinement. This study aimed to create an effective tool to enhance csPCa diagnosis and reduce unnecessary biopsies.

## 2 Methods

### 2.1 Study Population

From May 2022 to January 2025, we enrolled male patients with elevated PSA scheduled for prostate biopsy. Data were retrospectively collected from three Chinese medical centres: Tongji Hospital, Tongji Medical College, Huazhong University of Science and Technology; Changhai Hospital, Shanghai; and Fujian Union Hospital, Fujian Medical University. These data were subsequently randomly divided into a training and internal validation cohorts at a 7:3 ratio for model construction and validation. Additionally, data collected between January and March 2025 from two of these medical centres (Tongji Hospital, Tongji Medical College, Huazhong University of Science and Technology; and Fujian Union Hospital, Fujian Medical University) were used to form an external validation cohort.

The eligibility criteria for participant enrollment in this study were as follows: (1) men with elevated PSA; (2) candidates for their initial prostate biopsy; (3) the ability to provide 30–50 mL of first morning urine before the biopsy. Participants were excluded if they met any of the following conditions: (1) a history of prior prostate biopsy or PCa; (2) use of medications or hormonal therapies known to affect PSA levels within 3 to 6 months; (3) concurrent acute prostatitis; (4) a history of invasive treatment for BPH or lower urinary tract symptoms within 6 months; (5) hepatitis and/or human immunodeficiency virus (HIV) infection. All patients underwent standard prostate biopsy. At least two experienced pathologists reviewed all prostate biopsy specimens. CsPCa was defined as an International Society of Urological Pathology (ISUP) grade group (GG) ≥ 2 or a Gleason Score (GS) ≥ 7.

The study was conducted in accordance with the principles of the Declaration of Helsinki. Ethical approval was obtained from the institutional review boards of Tongji Hospital, Tongji Medical College, Huazhong University of Science and Technology (approval number: TJ‐IRB20210957); Changhai Hospital, Shanghai (approval number: CHEC2023‐180); and Fujian Union Hospital, Fujian Medical University (approval number: 2025KY267). Given that only anonymized retrospective data were utilized, the ethics committees waived the requirement for informed consent.

### 2.2 Sample Collection, EVs, and Total RNA Isolation, Characterization, PCa‐TARGET Genes Detection, and RT‐qPCR Data Analysis

Participants provided 30–50 mL of first morning urine before the biopsy procedure. DRE was avoided prior to urine collection to prevent contamination. The urine sample was centrifuged at 2000 × *g* for 20 min at 4°C to remove cellular debris. A total of 30 mL of supernatant was collected and processed using the EXODUS platform to isolate urinary EVs (Chen et al. 2021). The isolated urinary EVs were further characterized by nanoparticle tracking analysis (NTA) to determine their size distribution, validated for EV‐specific markers (Alix, TSG101, CD63, and CD9) via Western blot (WB), and examined for typical cup‐shaped morphological features using transmission electron microscopy (TEM). RNA extraction from EVs, RT‐qPCR, and analysis of target gene expression were conducted as described in our previously published study (Jiang et al. 2024). Details of target genes are listed in Table S1.

### 2.3 Screening of Urinary EVs Genes

We conducted a systematic review of EV‐derived gene signatures for PCa. Relevant studies on the target genes are listed in Table S2. To evaluate the predictive performance of these genes for csPCa, we used scikit‐learn (1.0.2) and LightGBM (4.5.0) in Python 3.7.16 to construct models and compare their predictive performance across 10 machine learning algorithms. These algorithms included AdaBoost, Bagging, Decision Tree, Gradient Boosting, k‐Nearest Neighbours (KNN), Logistic Regression, Multi‐Layer Perceptron (MLP), Support Vector Machine (SVM), Random Forest, and LightGBM. The top‐performing gene combination was subsequently selected to construct the final EGPS model.

### 2.4 Development and Evaluation of the EGPS Diagnostic Model

This study developed and validated the model through four steps. First, samples were randomly divided into training and internal validation cohorts at a 7:3 ratio. Candidate models were trained on the training cohort and evaluated using AUC on the internal validation cohort to select the optimal model. Second, the training and internal validation cohorts were stratified into five PSA‐based subgroups (0–10, 0–15, 0–20, 0–25, and 0–ALL ng/mL). Each subgroup was used to train multiple models, and their AUCs in corresponding internal validation cohorts were compared to identify the optimal PSA subgroup. Third, based on the optimal PSA subgroup, the EGPS model was constructed in the training cohort for csPCa prediction and validated in internal and external validation cohorts. To comprehensively evaluate model performance, we conducted comparative analyses between the EGPS model and other common indicators, including PSA and the clinical parameter model (CPM, which incorporates age and PSA). The DeLong test was used to assess the statistical significance of differences in AUC. Decision curve analysis (DCA) curves and waterfall plots of predicted probabilities were used to determine the clinical decision‐making value of the EGPS model across different risk thresholds and patient distributions. Fourth, the optimal cut‐off value was set to achieve a sensitivity greater than 95% for csPCa prediction. Based on the cut‐off value, sensitivity, specificity, missed diagnosis rate, and avoidable unnecessary biopsy rates were calculated to quantify the clinical value of the EGPS model.

### 2.5 Development and Application of an Online AI Diagnostic System

To support clinical decisions and provide real‐time interaction, we developed the EGPS online AI diagnosis system by integrating the EGPS model, Shapley Additive Explanations (SHAP) interpretability analysis, DeepSeek, and the optical character recognition (OCR) function from the Baidu AI Open Platform. When users input gene expression data, the system automatically loads the model using the joblib library (version 1.2.0), processes the data, computes the EGPS score, and classifies the predictions based on the predefined cut‐off value. It then outputs diagnosis and biopsy recommendations.

We used SHAP analysis to enhance the interpretability of the EGPS model by assessing the impact of each feature on the model's predictions. Global feature importance was assessed via summary plots and mean absolute SHAP values. SHAP force plots and individual diagrams showed the effects of local features on single‐patient predictions. Dependency and distribution plots revealed non‐linear relationships and inter‐patient heterogeneity. All SHAP analyses were performed using Python's SHAP package (version 0.42.1), using a Random Forest model as the base learner to estimate feature importance.

To offer personalized recommendations, the system uses the DeepSeek‐R1 application programming interface (API) for AI‐driven diagnostic and treatment suggestions. The system calibrates DeepSeek output using structured prompts based on the European Association of Urology (EAU) guidelines for PCa management, along with a standardized response format. By integrating patients' clinical characteristics, model predictions, and user queries, DeepSeek generates guideline‐compliant recommendations to provide personalized insights. The entire system is packaged into an executable (EXE) using the Tkinter library (version 0.1.0) in Python 3.7.16, deployed on Tencent Cloud servers, and accessible via designated IP addresses and ports.

## 3 Results

### 3.1 Patient Characteristics

A total of 645 patients met the inclusion and exclusion criteria. First, a study cohort of 586 patients was established at three medical centres between May 2022 and January 2025, and then divided into a training cohort (*n* = 410) and an internal validation cohort (*n* = 176). Subsequently, all 586 patients were stratified into PSA‐based groups: 0–10 ng/mL (*n* = 316), 0–15 ng/mL (*n* = 483), 0–20 ng/mL (*n* = 563), 0–25 ng/mL (*n* = 569) and 0–ALL ng/mL (encompassing all patients, *n* = 586). Subgroup details are provided in Tables S3–S7. No significant differences were observed between the training and validation cohorts (all *p* > 0.05). In addition, this study enrolled 59 eligible patients from two medical centres between January 2025 and March 2025 to establish an independent external validation cohort.

### 3.2 The Extraction and Characterization of EVs and Nucleic Acids

Figure S1 presents the NTA, WB, and TEM results of urinary EVs isolated via EXODUS. The NTA revealed a size distribution ranging from 30 to 300 nm, indicating adequate particle enrichment (Figure S1A). WB analysis confirmed the presence of key EV markers, such as CD9, CD63, TSG101, and Alix (Figure S1B). TEM imaging demonstrated their characteristic cup‐shaped morphology against a clean background (Figure S1C,D). Total RNA was automatically extracted from EVs using a magnetic bead‐based nucleic acid extraction method. The electropherogram of total RNA (Figure S2) exhibited two distinct peaks at approximately 25 and 50 nt, with a fragment length range of 20–2000 nt.

### 3.3 Gene Screening, Model Development, and Performance Evaluation

This study assessed eleven candidate genes, including AMACR, DLX1, FOXA1, HOXB13, KLK3, MALAT1, PCA3, PSCA, PSGR, PSMA, and USP9Y‐TTTY15. Among the 10 machine learning algorithms tested, the gene combination comprising AMACR, HOXB13, and PSGR achieved the best performance in diagnosing csPCa. This combination was used to construct the EGPS model. Subsequently, to optimize clinical application, model performance was evaluated across various PSA subgroups. As shown in Table S8, the Random Forest‐based EGPS model achieved the highest AUC in the PSA 0–15 ng/mL subgroup.

Subsequent analyses focused on the subgroup with PSA levels of 0–15 ng/mL, which comprised 483 patients (Table S9). This subgroup included 338 patients in the training cohort (Table S10) and 145 patients in the internal validation cohort (Table S11). Additionally, an external validation cohort of 59 patients was established (Table S12).

**FIGURE 2:**
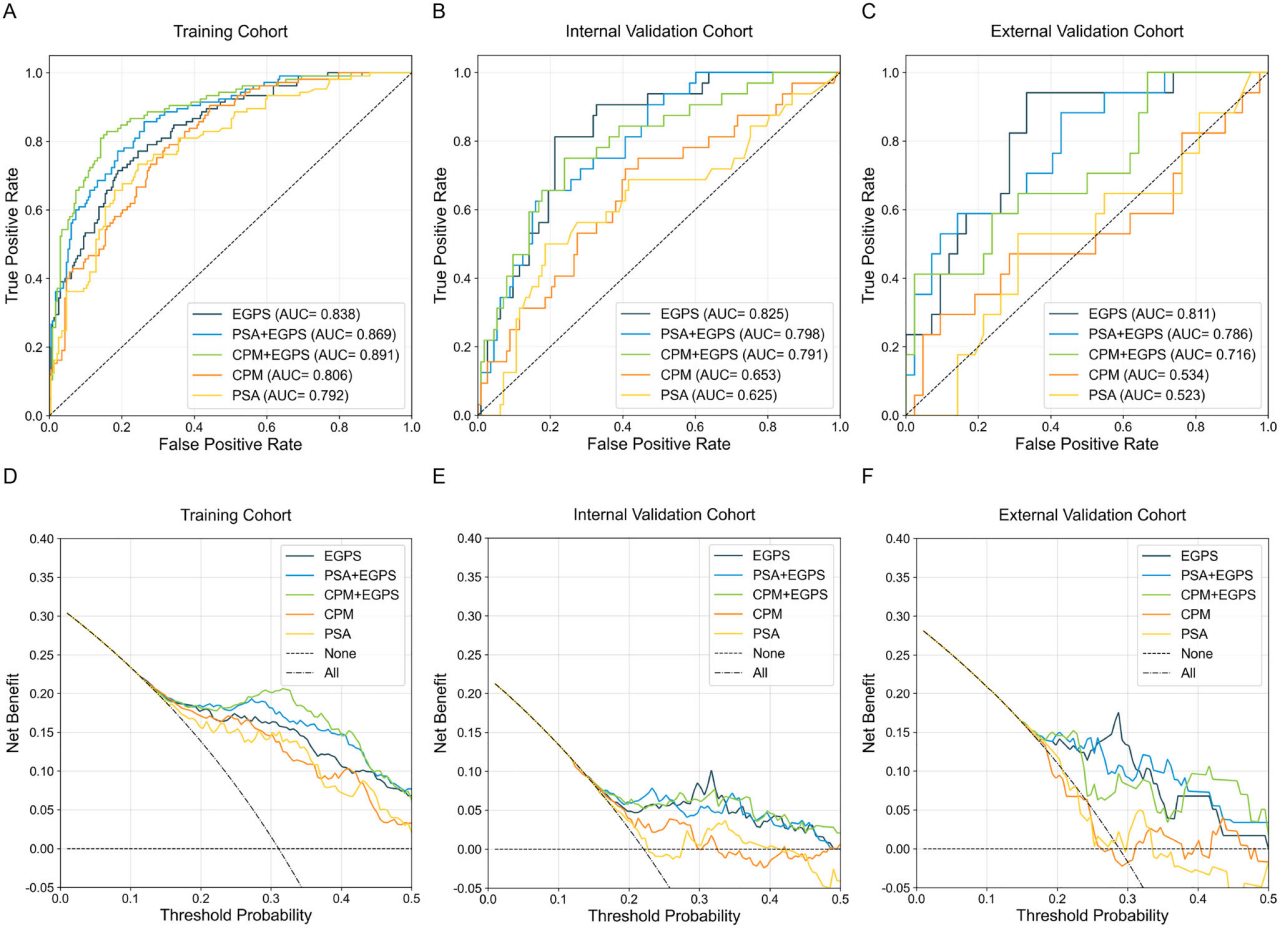
Evaluation of the models' performance and net benefit analysis of PSA 0–15 ng/mL. (A) Receiver Operating Characteristic (ROC) curves for predicting csPCa in the training cohort. (B) ROC curves for predicting csPCa in the internal validation cohort. (C) ROC curves for predicting csPCa in the external validation cohort. (D) Decision curve analysis (DCA) for csPCa prediction in the training cohort. (E) DCA for csPCa prediction in the internal validation cohort. (F) DCA for csPCa prediction in the external validation cohort. EGPS, Extracellular vesicles Gene‐based Prostate Score (including AMACR, HOXB13, and PSGR); PSA, prostate‐specific antigen; CPM, clinical parameter model (including age and PSA).

The EGPS model demonstrated an AUC of 0.838 for predicting csPCa in the training cohort, 0.825 in the internal validation cohort, and 0.811 in the external validation cohort, significantly outperforming independently applied PSA and CPM (Table 1, Figure 2A–C).

**TABLE 1:** The AUCs and Delong test of models for predicting csPCa in PSA 0–15 ng/mL under the random forest algorithm.

	Training cohort		Internal validation cohort		External validation cohort	
Models	AUC (95% CI)	*p* value	AUC (95% CI)	*p* value	AUC (95% CI)	*p* value
EGPS	0.838 (0.789–0.882)	0.190	0.825 (0.747–0.894)	0.005	0.811 (0.679–0.911)	0.003
PSA+EGPS	0.869 (0.825–0.910)	0.008	0.798 (0.718–0.874)	0.004	0.786 (0.650–0.900)	0.007
CPM+EGPS	0.891 (0.853–0.926)	0.002	0.791 (0.700–0.874)	0.010	0.716 (0.561–0.855)	0.065
CPM	0.806 (0.761–0.851)	0.608	0.653 (0.536–0.760)	0.578	0.534 (0.346–0.713)	0.923
PSA	0.792 (0.739–0.843)	—	0.625 (0.501–0.736)	—	0.523 (0.344–0.688)	—

Abbreviations: AUC, area under curve; CPM, clinical parameter model (including age and PSA); csPCa, clinical significantly prostate cancer; EGPS, Extracellular vesicles Gene‐based Prostate Score (including AMACR, HOXB13, and PSGR); PSA, total prostate‐specific antigen.

DCA was employed to compare the net benefit of the EGPS model with standard clinical strategies across different probability thresholds (Figure 2D–F). The DCA results from the training, internal, and external validation cohorts demonstrated that within the risk threshold range of 0.1–0.5, the EGPS model provided a higher net benefit for predicting csPCa than the treat‐all or treat‐none strategies.

### 3.4 EGPS Model Enhances Sensitivity While Reducing Unnecessary Biopsies

To reduce unnecessary biopsies and minimize missed diagnoses, the EGPS model's cut‐off value for csPCa prediction was set to 0.22, ensuring >95% sensitivity (Table 2). Patients with an EGPS score >0.22 were predicted to have csPCa. In the training cohort, sensitivity reached 96.19% for predicting csPCa with only four missed cases (3.81%). No missed diagnoses were observed in the internal or external validation cohorts. Furthermore, unnecessary prostate biopsies were avoided in 79 patients (23.37%), 27 patients (18.62%), and 9 patients (15.25%) in the training, internal validation, and external validation cohorts, respectively. Figure S3 illustrates the EGPS model's performance at the cut‐off value of 0.22 using predicted probability waterfall plots across all cohorts. These findings indicate that the EGPS model can accurately identify csPCa while limiting overtreatment, demonstrating promising prospects for clinical application.

**TABLE 2:** EGPS model performance in predicting csPCa of PSA 0–15 ng/mL at a cut‐off value of 0.22.

Evaluating model performance in predicting csPCa at a 0.22 cut‐off value												
	Training cohort				Internal validation cohort				External validation cohort			
	EGPS ≥ cut‐off value	EGPS < cut‐off value	Total	Performance, %	EGPS ≥ cut‐off value	EGPS < cut‐off value	Total	Performance, %	EGPS ≥ cut‐off value	EGPS < cut‐off value	Total	Performance, %
CsPCa	101	4	105	Sensitivity, 96.19	32	0	32	Sensitivity, 100.00	17	0	17	Sensitivity, 100.00
Non‐csPCa	158	75	233	Specificity, 32.19	86	27	113	Specificity, 23.89	33	9	42	Specificity, 21.43
Total	259	79	338	PPV, 39.00	118	27	145	PPV, 27.12	50	9	59	PPV, 34.00
				NPV, 94.94				NPV, 100.00				NPV, 100.00
Predicted negative	23.37%				18.62%				15.25%			
Missed diagnosis	3.81%				0.00%				0.00%			

Abbreviations: csPCa, clinically significant prostate cancer; EGPS, Extracellular vesicles Gene‐based Prostate Score (including AMACR, HOXB13, and PSGR); NPV, negative predictive value; PPV, positive predictive value.

### 3.5 EGPS Online AI Diagnostic Consultation System

**FIGURE 3:**
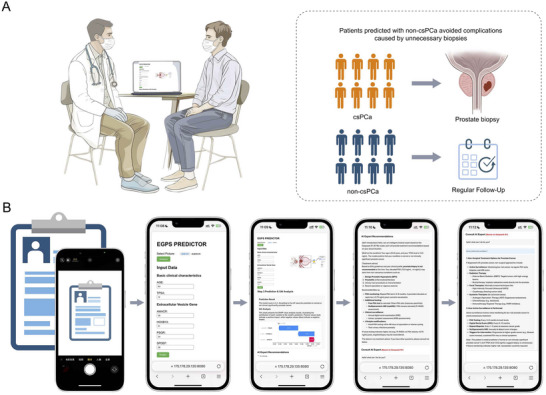
Workflow of the EGPS artificial intelligence diagnostic system for csPCa risk assessment. (A) Illustration of the clinical application of the EGPS AI system. Patients predicted as having clinically significant prostate cancer (csPCa) are referred for biopsy. In contrast, those indicated as non‐csPCa are recommended for regular follow‐up, thereby avoiding unnecessary procedures and associated complications. (B) Workflow of the EGPS online AI system. Users upload patient data via OCR or manual input, including clinical parameters and urinary EV gene expression. The system automatically computes the EGPS score, visualizes model interpretation via SHAP analysis, and provides expert‐level recommendations powered by DeepSeek, enabling individualized, informed clinical decision‐making. EGPS, Extracellular vesicles Gene‐based Prostate Score (including AMACR, HOXB13, and PSGR).

The EGPS online AI diagnostic system is available at: http://175.178.29.135:8080. A 63‐year‐old man with a PSA of 13.0 ng/mL was used to illustrate this application (Figure 3A). According to guidelines (Edn 2023), this patient would typically be referred for a prostate biopsy. His urinary EVs gene expression levels were as follows: AMACR = 28, HOXB13 = 21, PSGR = 34, and SPDEF = 28. After inputting his clinical information and urinary EV gene data, the system output a score of 0.2 (below the 0.22 threshold), indicating non‐csPCa. The system recommended follow‐up monitoring instead of immediate biopsy to avoid an unnecessary intervention (Figure 3B).

SHAP‐based XAI (Explainable Artificial Intelligence) analysis was employed to interpret the EGPS model within the PSA 0–15 ng/mL subgroup (Figure S4). Ultimately, the SHAP analysis module was embedded in the EGPS system as a waterfall plot for intuitive visualization of feature impact. For this patient, PSGR had the most positive impact on the final csPCa prediction (SHAP value = +0.16). HOXB13 and AMACR had slight negative contributions (SHAP values = −0.03 and −0.01) on csPCa prediction (Figure 3B).

After predictions, the DeepSeek AI generates clinical summaries and personalized treatment recommendations (Figure 3B). An AI‐powered interactive Q&A function was integrated, enabling users to submit PCa‐related questions directly to the DeepSeek model. It offers real‐time expert‐level answers on diagnosis, treatment, and follow‐up of PCa.

## 4 Discussion

Although PSA is widely used for PCa screening, its limited specificity for csPCa often causes overdiagnosis and unnecessary biopsies. To overcome this limitation, several studies have focused on developing blood‐based detection methods for csPCa diagnosis. However, their clinical application is somewhat limited due to the need for invasive sample collection. This has spurred the development of more convenient and non‐invasive urine‐based detection methods, which are currently a research hotspot. In 2019, Select MDx targets patients with PSA ≥ 3 ng/mL and predicts csPCa using HOXC6 and DLX1 mRNA in whole urine samples (Van Neste et al. 2016). Additionally, the EPI predicts the risk of csPCa by analysing PCA3, ERG, and SPDEF in urinary EVs for patients with PSA 2–10 ng/mL (Donovan et al. 2015; McKiernan et al. 2016). However, these models haven't systematically evaluated the differences across different PSA subgroups. This study, using urine as the basis for detection, innovatively stratified patients into five PSA‐based subgroups and conducted stratified validation. The results showed that the EGPS model, constructed using urinary EVs in this study, exhibited optimal diagnostic efficacy for csPCa in patients with PSA levels of 0–15 ng/mL. This model not only overcame the diagnostic limitations of traditional models in the PSA grey zone but also further improved the predictive efficacy of csPCa.

The non‐invasive nature and accessibility of urine make it an ideal specimen for csPCa screening. Numerous studies have used post‐DRE urine to develop predictive models for csPCa. For instance, Kotova et al. developed a predictive model by analysing the mRNA expression of AMACR and PCA3 in post‐DRE whole urine, combined with PSA, with an AUC of 0.72 for csPCa (Kotova et al. 2020). Tomlins et al. constructed a multivariable MiPS model using T2: ERG and PCA3 mRNA from post‐DRE urine combined with PSA, which demonstrated an AUC of 0.751 (Tomlins et al. 2016). However, DRE reduces patient comfort and sampling standardization (Romero et al. 2008). Consequently, current research has shifted toward completely non‐invasive approaches that do not require DRE. Urinary EVs can stably carry high‐quality RNA, enabling the development of PCa diagnostic models using non‐DRE urine (Jin et al. 2022; Tao et al. 2023). Several studies have successfully developed urine EVs‐based models for predicting csPCa. Ramirez‐Garrastacho et al. (2022) used miR‐155‐5p/miR‐320a‐3p from non‐DRE urine EVs to build a model for csPCa, achieving an AUC of 0.76. McKiernan et al. developed the EPI model using non‐DRE urine EVs, yielding an AUC of 0.73 in the validation cohorts for csPCa detection (McKiernan et al. 2016). However, these models were derived from Western populations and have not been validated in Chinese cohorts. Notably, several non‐DRE‐based urinary biomarkers, including proteomic panels and collagen‐derived peptide assays, have already been validated in recent multicentre studies (Frantzi et al. 2019; Frantzi et al. 2024; Frantzi et al. 2025), whereas the present EGPS model adopts a more methodologically streamlined approach based on targeted urinary EV gene expression, which may be easier to implement in routine clinical practice. This multicentre Chinese study developed the EGPS model using three clinically validated biomarkers (AMACR, HOXB13 and PSGR) derived from non‐DRE urine EVs. In Chinese patients with PSA 0–15 ng/mL, the EGPS model demonstrated AUCs of 0.838, 0.825, and 0.811 in the training, internal, and external validation cohorts, respectively, outperforming PSA. These findings highlight that the EGPS model, based on non‐DRE urinary EV biomarkers, offers a promising, high‐accuracy tool for csPCa screening in Chinese patients with PSA levels of 0–15 ng/mL.

While PSA remains the most widely used biomarker for PCa screening, clinical decisions based on PSA led to unnecessary prostate biopsies in over 50% of patients (Roddam et al. 2005). This exposes patients without PCa to biopsy‐related complications and increases medical and economic burdens (Fenton et al. 2018; Heijnsdijk et al. 2012). To address these limitations, extensive research has focused on developing alternatives to PSA. Filella et al. (2014) demonstrated that blood‐based biomarkers incorporating the PHI, percentage of prostate‐specific antigen precursor (%p2PSA), and percentage of free PSA (%fPSA) could reduce unnecessary biopsies by 19%, 12.7%, and 13.8%, with corresponding csPCa missed diagnosis rates of 5.0%, 5.0%, and 8.8%, respectively. McKiernan et al. (2018) further reported that the urinary EV‐based EPI model avoided 20% of unnecessary biopsies at the cost of a 7% missed detection rate of csPCa. However, these models lack validation in Chinese populations. This study developed the EGPS model for Chinese patients with PSA 0–15 ng/mL, resulting in reduction of 23.37%, 18.62%, and 15.25% in unnecessary biopsies for the training, internal validation, and external validation cohorts, respectively. Meanwhile, the missed diagnosis rate was 3.81% in the training cohort, with no missed diagnoses in the internal or external validation cohorts, demonstrating that the strong clinical utility of EGPS model for predicting csPCa. In summary, the EGPS model, based on non‐DRE urinary EV genes, reduces the overdiagnosis and underdiagnosis of csPCa in Chinese patients, thereby alleviating the medical and financial burdens on patients. It complements PSA screening and supports precise diagnostic and treatment decisions for csPCa.

AI has rapidly expanded in clinical practice, especially with large language models transforming healthcare. China's DeepSeek models have demonstrated strong clinical adaptability through deep learning (Sandmann et al. 2025). Based on DeepSeek‐R1, this study developed the EGPS intelligent diagnostic system to predict csPCa accurately. It features an interactive Q&A function that provides patients with real‐time, tailored recommendations regarding PCa management. A SHAP module embedded in the system enhances model interpretability by visualizing each feature's contribution to predictions, thereby enhancing clinical usability and acceptance among clinicians and patients.

This study is subject to several limitations. First, as a retrospective study, the findings have not been validated in real‐world clinical settings and warrant confirmation through prospective studies. Second, the model is optimized for patients with PSA levels of 0‐ 15 ng/mL and may require adjustments for higher PSA values. Third, the clinical usability of the AI‐driven Q&A module has not been systematically evaluated.

## 5 Conclusion

This multicentre Chinese study established a csPCa predictive model based on the AMACR, HOXB13, and PSGR genes derived from EVs isolated from non‐DRE urine via the EXODUS method. Additionally, the EGPS online AI diagnostic system was developed with DeepSeek. This system provides accurate and interpretable predictions of csPCa for first‐time biopsy candidates with PSA levels ranging from 0 to 15 ng/mL. By effectively reducing unnecessary biopsies, it represents a safe and effective tool for early csPCa screening and supports personalized clinical decision‐making.

## Author Contributions

**Shaoqin Jiang**: investigation, funding acquisition, writing – original draft, writing – review and editing. **Chunguang Yang**: conceptualization, investigation. **Zhangcheng Huang**: investigation. **Zebang Guo**: investigation. **Feiting Lu**: investigation, writing – original draft, funding acquisition. **Xinwen Nian**: methodology. **Zhenlin Chen**: methodology, validation. **Pengwei Luo**: methodology, validation. **Jiawei Jiang**: methodology, validation. **Xu Gao**: conceptualization, investigation, resources, funding acquisition. **Mengqiang Li**: conceptualization, investigation, resources, funding acquisition. **Fei Liu**: conceptualization, investigation, supervision.

## Conflicts of Interest

The authors declare no conflicts of interest.

## Supporting information




**Supplementary Materials**: jev270233‐sup‐0001‐Appendix.pdf

## Data Availability

The datasets used in this study are not publicly available but can be shared in de‐identified form upon reasonable request to the corresponding author.
