# Supplementary material for: Multicentre Evaluation of an AI‐Assisted Urine Test for Clinically Significant Prostate Cancer in Men Undergoing Initial Biopsy
Source: J Extracell Vesicles. 2026 Mar 25;15(4):e70233. doi: 10.1002/jev2.70233 (PMC13140973; doi:10.1002/jev2.70233)
Supplement: Supplementary file 1 — Supplementary Materials: jev270233‐sup‐0001‐Appendix.pdf [file JEV2-15-e70233-s001.pdf]

Supplementary Content

Table S1 Design Information for Primers and Probes.. 2
Table S2 Compilation of Key Research Articles on EVs Genes Linked to Prostate Cancer. 5
Table S3 Clinical Characteristics of the Training and Internal Validation Cohorts in tPSA 0-10 ng/mL. 7
Table S4 Clinical Characteristics of the Training and Internal Validation Cohorts in tPSA 0-15 ng/mL. 9
Table S5 Clinical Characteristics of the Training and Internal Validation Cohorts in tPSA 0-20 ng/mL. 11
Table S6 Clinical Characteristics of the Training and Internal Validation Cohorts in tPSA 0-25 ng/mL. 13
Table S7 Clinical Characteristics of the Training and Internal Validation Cohorts in tPSA 0- All ng/mL. 15
Table S8 AUC (95% CI) of Models for Predicting csPCa of Urinary EVs Genes and Prostate Cancer-Associated Clinical Features in Patients of Different tPSA Levels... 17
Table S9 Clinical Characteristics of the Cohorts in tPSA 0-15 ng/mL Used to Train and Validate the Model. 18
Table S10 Clinical Characteristics of the Training Cohorts in tPSA 0-15 ng/mL.. 19
Table S11 Clinical Characteristics of the Internal Validation Cohorts in tPSA 0-15 ng/mL. 21
Table S12 Clinical Characteristics of the External Validation Cohort in tPSA 0-15 ng/mL... 23
Figure S1. Characterization of EVs isolated from urine. 25
Figure S2. The electrophoresis chromatogram of total RNA analyzed by Agilent Bioanalyzer 2100. 26
Figure S3. Waterfall plots of predicted probabilities for csPCa in different cohort using the EGPS model. 28
Figure S4. XAI analysis of model in predicting csPCa of tPSA 0-15 ng/mL. 30

Table S1 Design Information for Primers and Probes.

Table S1. Design Information for Primers and Probes.*					
**Genes**	**NCBI sequence**	**Amplification length**	**Primer/Probe**	**sequence**	**Working concentration**
AMACR	NM_014324. NM_001167595.2 NM_203382.3	98 bp	AMACR Forward primer	AATGAAGAAGAAGTTTGCAGATGTA	300 nm
			AMACR Reverse primer	TCAGAACCGGAGTCACACA	300 nm
			AMACR Probe	HEX-TGCAGAAAAGACGAAGGCAGAGTGGTG-BHQ2	100 nm
DLX1	NM_178120.5 NM_001038493.2 XM_054340886.1	98 bp	DLX1 Forward primer	TCAGCTTTACAACAATCCCTA	300 nm
			DLX1 Reverse primer	CAGTCATTAGATCCTGCA	300 nm
			DLX1 Probe	FAM-ACCACAGAATAATGCCAGTCACCAC-BHQ1	100 nm
FOXA1	NM_004496.5	132 bp	FOXA1 Forward primer	GGCATGAAACCAGCGACT	300 nm
			FOXA1 Reverse primer	TGGTGTTCATGGTCATGTAGGT	300 nm
			FOXA1 Probe	ROX-CCCGGTCAGCAACATGAACTCAGG-BHQ2	100 nm
HOXB13	NM_006361.6	74 bp	HOXB13 Forward primer	CTCTCGGAGCGCCAGATTAC	300 nm
			HOXB13 Reverse primer	TTCACCTTGGCGAGAACCTT	300 nm
			HOXB13 Probe	FAM-TTTCAGAACCGCCGGGTCAAAGA-BHQ1	100 nm
KLK3	NM_001648.2 NM_001030047.1 NM_001030048.1	89 bp	KLK3 Forward primer	GAGAGCTGTGTCACCATG	400 nm
			KLK3 Reverse primer	ATCCGAGACAGGATGAGG	400 nm
			KLK3 Probe	HEX-AGCACCAATCCACGTCACGGACAG-BHQ2	200 nm

MALAT1	NR_002819.4 NR_144567.1 NR_144568.1	78 bp	MALAT1 Forward primer	CCCCGTGCCTTTTGATCTAG	400 nm
			MALAT1 Reverse primer	CATGCCCACAAGGATCCAA	400 nm
			MALAT1 Probe	FAM-CCCCTCACCTCGATGCAGCCA-BHQ1	150 nm
PCA3	NR_132312.1 NR_015342.2 NR_132313.1	135 bp	PCA3 Forward primer	GCTGGAAATGGACAACCAC	300 nm
			PCA3 Reverse primer	CCAGCTGAGACCTAATGCAAG	300 nm
			PCA3 Probe	FAM-ACCCACAAATGCGAGGTGCTTC-BHQ1	150 nm
PSCA	NR_033343.2 NM_005672.5	95 bp	PSCA Forward primer	ATCGGCTCTATTGACACAGATCC	300 nm
			PSCA Reverse primer	ACAGGGTTAAGGGTGGAGAATG	300 nm
			PSCA Probe	HEX-CCCTCCAACCCTCTCTGCTGCTGTTTCCAT-BHQ1	100 nm
PSGR	NM_030774.4	106 bp	PSGR Forward primer	CTGAGTCAGCCTGAAAGGAACA	300 nm
			PSGR Reverse primer	CAGGAACTCATAGCTGGAACTGA	300 nm
			PSGR Probe	ROX-TCTACTGCCAGTGTGACCTCACCCTCTCCA-BHQ2	100 nm
PSMA	NM_004476.3 NM_001014986.3 NM_001193471.3 NM_001193472.3 NM_001193473.3 NM_001351236.2	104 bp	PSMA Forward primer	TGCACAGAAGCTCCTAGAAAAAATG	300 nm
			PSMA Reverse primer	TTCCAGTAAAGCCAGGTCCAA	300 nm
			PSMA Probe	CY5-TGGCTCAGCACCACCAGATAGCAGC-BHQ2	100 nm
SPDEF	NM_012391.3 NM_001252294.2	99 bp	SPDEF Forward primer	GGCGAAGTGCTCAAGGACAT	400 nm

			SPDEF Reverse primer	CAGGAGCCACTTCTGCACATT	400 nm
			SPDEF Probe	CY5-ACGGCCTGCAAGCTGCTCAACATC-BHQ2	200 nm
USP9Y-TTTY15	NM_004654.4 XM_047442772.1 XM_047442771.1 XM_054328378.1 XM_054328377.1 NR_001545.3 NR_174087.1 NR_174088.1 NR_174085.1 NR_174086.1	163 bp	USP9Y-TTTY15 Forward primer	CCCTTGGAAAAACTGGCCTCATA	300 nm
			USP9Y-TTTY15 Reverse primer	GCCTATCAATTGCTGCCTTATATA	300 nm
			USP9Y-TTTY15 Probe	HEX-ACACATACTCCACACAGCCACCAGAAT-BHQ2	100 nm

* AMACR, α-methylacyl-CoA racemase; DLX1, distal-less homeobox 1; FOXA1, fork-head box 1; HOXB13, homeobox B13; KLK3, kallikrein related peptidase 3; MALAT1, metastasis-associated lung adenocarcinoma transcript 1; PCA3, prostate cancer antigen 3; PSCA, prostate stem cell antigen; PSGR, prostate-specific G-protein coupled receptor; PSMA, prostate-specific membrane antigen; SPDEF, SAM pointed domain containing ETS transcription factor; USP9Y-TTTY15, ubiquitin specific peptidase 9, Y-linked, testis-specific transcript, Y-linked 15.

Table S2 Compilation of Key Research Articles on EVs Genes Linked to Prostate Cancer.

Table S2. Compilation of Key Research Articles on EVs Genes Linked to Prostate Cancer.*	
**Genes**	**Articles about Urinary EVs genes with prostate cancer**
AMACR	Kotova ES, Savochkina YA, Doludin YV, et al. Identification of Clinically Significant Prostate Cancer by Combined PCA3 and AMACR mRNA Detection in Urine Samples. Res Rep Urol 2020;Volume 12:403–13.
	Jiang N, Zhu S, Chen J, Niu Y, Zhou L. A-methylacyl-CoA racemase (AMACR) and prostate-cancer risk: a meta-analysis of 4,385 participants. PLoS One 2013;8(10):e74386.
DLX1	Goel S, Bhatia V, Kundu S, et al. Transcriptional network involving ERG and AR orchestrates Distal-less homeobox-1 mediated prostate cancer progression. Nat Commun 2021;12(1):5325.
	Liang M, Sun Y, Yang H-L, Zhang B, Wen J, Shi B-K. DLX1, a binding protein of beta-catenin, promoted the growth and migration of prostate cancer cells. Exp Cell Res 2018;363(1):26–32.
FOXA1	Zhao JC, Fong K-W, Jin H-J, Yang YA, Kim J, Yu J. FOXA1 acts upstream of GATA2 and AR in hormonal regulation of gene expression. Oncogene 2016;35(33):4335–44.
	Jeter CR, Liu B, Lu Y, et al. NANOG reprograms prostate cancer cells to castration resistance via dynamically repressing and engaging the AR/FOXA1 signaling axis. Cell Discov 2016;2:16041.
HOXB13	Kim Y-R, Kang TW, To PK, et al. HOXB13-mediated suppression of p21WAF1/CIP1 regulates JNK/c-Jun signaling in prostate cancer cells. Oncol Rep 2016;35(4):2011–6.
	Danila DC, Samoila A, Patel C, et al. Clinical Validity of Detecting Circulating Tumor Cells by AdnaTest Assay Compared With Direct Detection of Tumor mRNA in Stabilized Whole Blood, as a Biomarker Predicting Overall Survival for Metastatic Castration-Resistant Prostate Cancer Patients. Cancer J 2016;22(5):315–20.
KLK3	Sawada T, Nishimura K, Mori J, et al. Androgen-dependent and DNA-binding-independent association of androgen receptor with chromatic regions coding androgen-induced noncoding RNAs. Biosci, Biotechnol, Biochem 2021;85(10):2121–30.
	Hsieh C-L, Fei T, Chen Y, et al. Enhancer RNAs participate in androgen receptor-driven looping that selectively enhances gene activation. Proc Natl Acad Sci U S A 2014;111(20):7319–24.
MALAT1	Goyal B, Yadav SRM, Awasthee N, Gupta S, Kunnumakkara AB, Gupta SC. Diagnostic, prognostic, and therapeutic significance of long non-coding RNA MALAT1 in cancer. Biochim Biophys Acta (BBA) - Rev Cancer 2021;1875(2):188502.
	Ren S, Liu Y, Xu W, et al. Long noncoding RNA MALAT-1 is a new potential therapeutic target for castration resistant prostate cancer. J Urol 2013;190(6):2278–87.
PCA3	Lee D, Shim SR, Ahn ST, et al. Diagnostic Performance of the Prostate Cancer Antigen 3 Test in Prostate Cancer: Systematic Review and Meta-analysis. Clin Genitourin Cancer 2020;18(5):402-408.e5.
	Ploussard G, de la Taille A. The role of prostate cancer antigen 3 (PCA3) in prostate cancer detection. Expert Rev Anticancer Ther 2018;18(10):1013–20.
PSCA	Nayerpour Dizaj T, Doustmihan A, Sadeghzadeh Oskouei B, et al. Significance of PSCA as a novel prognostic marker and therapeutic target for cancer. Cancer Cell Int 2024;24(1):135.
	Li E, Liu L, Li F, et al. PSCA promotes prostate cancer proliferation and cell-cycle progression by up-regulating c-Myc. Prostate 2017;77(16):1563–72.
PSGR	Rodriguez M, Siwko S, Liu M. Prostate-Specific G-Protein Coupled Receptor, an Emerging Biomarker Regulating Inflammation and Prostate Cancer Invasion. Curr Mol Med 2016;16(6):526–32.
	Cao W, Li F, Yao J, Yu J. Prostate specific G protein coupled receptor is associated with prostate cancer prognosis and affects cancer cell proliferation and invasion. BMC Cancer 2015;15:915.
PSMA	Bakht MK, Yamada Y, Ku S-Y, et al. Landscape of prostate-specific membrane antigen heterogeneity and regulation in AR-positive and AR-negative metastatic prostate cancer. Nat Cancer 2023;4(5):699–715.
	Caromile LA, Dortche K, Rahman MM, et al. PSMA redirects cell survival signaling from the MAPK to the PI3K-AKT pathways to promote the progression of prostate cancer. Sci Signal 2017;10(470):eaag3326.
USP9Y-TTTY15	Zhu Y, Ren S, Jing T, et al. Clinical utility of a novel urine-based gene fusion TTTY15-USP9Y in predicting prostate biopsy outcome. Urol Oncol 2015;33(9):384.e9-20.

	Ren S, Peng Z, Mao J-H, et al. RNA-seq analysis of prostate cancer in the Chinese population identifies recurrent gene fusions, cancer-associated long noncoding RNAs and aberrant alternative splicings. Cell Res 2012;22(5):806–21.

* EVs, extracellular vesicles; AMACR, α-methylacyl-CoA racemase; DLX1, distal-less homeobox 1; FOXA1, fork-head box 1; HOXB13, homeobox B13; KLK3, kallikrein related peptidase 3; MALAT1, metastasis-associated lung adenocarcinoma transcript 1; PCA3, prostate cancer antigen 3; PSCA, prostate stem cell antigen; PSGR, prostate-specific G-protein coupled receptor; PSMA, prostate-specific membrane antigen; USP9Y-TTTY15, ubiquitin specific peptidase 9, Y-linked, testis-specific transcript, Y-linked 15.

Table S3 Clinical Characteristics of the Training and Internal Validation Cohorts in tPSA 0-10 ng/mL.

Table S3. Clinical Characteristics of the Training and Internal Validation Cohorts in tPSA 0-10 ng/mL.*				
	Median (IQR)			
Characteristic	Overall (n=316)	Training Cohort (n=221)	Internal Validation Cohort (n=95)	*P* value†
Age — yr	66.00 (61.00-70.00)	66.00 (61.00-71.00)	66.00 (61.00-69.50)	0.611
tPSA — ng/mL	6.67 (5.34-8.15)	6.66 (5.40-8.19)	6.69 (5.24-8.05)	0.982
AMACR	849.98 (438.53-1972.83)	868.60 (420.45-1905.28)	718.97 (456.82-2182.83)	0.871
DLX1	131.92 (22.64-1023.79)	153.79 (25.06-1005.56)	76.22 (17.48-1026.48)	0.289
FOXA1	2844.29 (2048.85-4840.38)	2922.12 (2048.95-5050.98)	2745.44 (2039.42-4507.97)	0.524
HOXB13	1376.00 (940.83-2178.74)	1365.26 (957.21-2200.77)	1386.67 (932.59-2112.79)	0.767
KLK3	866.34 (486.66-1292.90)	836.25 (476.34-1299.90)	917.00 (500.91-1265.39)	0.329
MALAT1	207.18 (67.46-1133.00)	219.16 (67.45-1324.49)	146.09 (68.28-1060.50)	0.462
PCA3	64.28 (20.99-333.14)	68.16 (20.93-365.57)	60.46 (24.59-283.17)	0.975
PSCA	5648.43 (2322.15-16274.19)	5715.98 (2291.04-16245.21)	5287.39 (2417.74-16395.13)	0.860
PSGR	50.10 (24.88-87.06)	50.17 (24.85-85.56)	49.22 (26.21-90.67)	0.771
PSMA	894.64 (592.01-1421.21)	892.55 (581.96-1452.97)	897.51 (604.37-1358.55)	0.979
USP9Y-TTTY15	13.39 (6.30-29.98)	12.05 (6.13-28.50)	15.89 (6.99-34.61)	0.467
Center — No. (%)				
Center1	161 (50.9%)	124 (56.1%)	37 (38.9%)	
Center2	87 (27.5%)	61 (27.6%)	26 (27.4%)	
Center3	68 (21.5%)	36 (16.3%)	32 (33.7%)	
Gleason Grade — No. (%)				0.353
Benign	214 (67.7%)	157 (71.0%)	57 (60.0%)	
GG 1 (GS 3 + 3)	27 (8.5%)	18 (8.1%)	9 (9.5%)	
GG 2 (GS 3 + 4)	42 (13.3%)	26 (11.8%)	16 (16.8%)	
GG 3 (GS 4 + 3)	16 (5.1%)	11 (5.0%)	5 (5.3%)	
GG 4 (all GS 8)	10 (3.2%)	6 (2.7%)	4 (4.2%)	
GG 5 (>GS 8)	7 (2.2%)	3 (1.4%)	4 (4.2%)	

* Values are median (IQR) unless indicated otherwise. IQR, interquartile range; tPSA, total prostate-specific antigen; Center1, Tongji Hospital, Tongji Medical College, Huazhong University of Science and Technology; Center2, Changhai Hospital, Shanghai; Center3, Fujian Union Hospital, Fujian Medical University; GG, Gleason Grade; GS, Gleason score.

† The *P* values are two sided, with no adjustment for multiple comparison.

Table S4 Clinical Characteristics of the Training and Internal Validation Cohorts in tPSA 0-15 ng/mL.

Table S4. Clinical Characteristics of the Training and Internal Validation Cohorts in tPSA 0-15 ng/mL.*				
	Median (IQR)			
**Characteristic**	**Overall (n=483)**	**Training Cohort (n=338)**	**Internal Validation Cohort (n=145)**	***P* value**[†]
Age — yr	66.00 (61.00-71.00)	66.00 (61.00-71.75)	66.00 (61.00-71.00)	0.521
tPSA — ng/mL	8.25 (6.07-11.50)	8.07 (6.07-11.53)	8.62 (6.06-11.45)	0.880
AMACR	844.43 (433.18-1978.66)	870.84 (427.98-1966.17)	737.93 (440.20-2020.90)	0.718
DLX1	133.27 (23.50-958.64)	119.71 (22.00-921.65)	174.82 (26.15-1122.41)	0.508
FOXA1	2783.88 (2066.23-4743.68)	2776.00 (2047.29-4763.62)	2785.54 (2091.64-4707.62)	0.772
HOXB13	1325.93 (959.09-2083.33)	1332.86 (953.78-2071.09)	1307.99 (993.92-2078.83)	0.503
KLK3	872.68 (483.89-1318.14)	917.00 (483.67-1386.79)	752.50 (486.66-1188.36)	0.089
MALAT1	208.05 (66.86-1097.29)	205.07 (63.55-1110.25)	216.51 (73.50-1073.26)	0.782
PCA3	68.50 (25.26-334.91)	65.60 (25.25-292.46)	89.87 (25.56-388.91)	0.648
PSCA	5445.26 (2267.10-16078.06)	5432.89 (2186.71-16188.39)	5532.76 (2389.98-16068.46)	0.698
PSGR	47.48 (25.66-87.42)	47.46 (25.55-84.41)	47.58 (25.92-92.78)	0.875
PSMA	893.72 (612.60-1385.87)	851.16 (598.20-1389.10)	1010.03 (632.89-1371.51)	0.145
USP9Y-TTTY15	12.79 (6.39-29.44)	13.60 (6.44-30.92)	11.79 (6.32-27.20)	0.236
Center — No. (%)				
Center1	254 (52.6%)	181 (53.6%)	73 (50.3%)	
Center2	135 (28.0%)	93 (27.5%)	42 (29.0%)	
Center3	94 (19.5%)	64 (18.9%)	30 (20.7%)	
Gleason Grade — No. (%)				0.073
Benign	304 (62.9%)	207 (61.2%)	97 (66.9%)	
GG 1 (GS 3 + 3)	42 (8.7%)	26 (7.7%)	16 (11.0%)	
GG 2 (GS 3 + 4)	67 (13.9%)	52 (15.4%)	15 (10.3%)	
GG 3 (GS 4 + 3)	37 (7.7%)	32 (9.5%)	5 (3.4%)	
GG 4 (all GS 8)	20 (4.1%)	14 (4.1%)	6 (4.1%)	
GG 5 (>GS 8)	13 (2.7%)	7 (2.1%)	6 (4.1%)	

* Values are median (IQR) unless indicated otherwise. IQR, interquartile range; tPSA, total prostate-specific antigen; Center1, Tongji Hospital, Tongji Medical College, Huazhong University of Science and Technology; Center2, Changhai Hospital, Shanghai; Center3, Fujian Union Hospital, Fujian Medical University; GG, Gleason Grade; GS, Gleason score.

† The *P* values are two sided, with no adjustment for multiple comparison.

Table S5 Clinical Characteristics of the Training and Internal Validation Cohorts in tPSA 0-20 ng/mL.

Table S5. Clinical Characteristics of the Training and Internal Validation Cohorts in tPSA 0-20 ng/mL.*				
	Median (IQR)			
Characteristic	Overall (n=563)	Training Cohort (n=394)	Internal Validation Cohort (n=169)	*P* value†
Age — yr, mean (±SD)	66.47 ± 7.76	66.66 ± 7.97	66.01 ± 7.27	0.341
tPSA — ng/mL	9.09 (6.40-13.11)	9.10 (6.42-13.03)	9.00 (6.34-13.31)	0.941
AMACR	868.59 (429.64-2007.02)	849.98 (407.56-1990.50)	877.14 (510.86-2101.74)	0.412
DLX1	142.40 (23.81-1000.02)	129.64 (22.79-1066.27)	147.30 (25.06-897.50)	0.755
FOXA1	2783.88 (2048.75-4821.58)	2747.94 (2045.33-4730.52)	2963.89 (2077.72-5173.88)	0.426
HOXB13	1340.63 (965.00-2083.33)	1354.27 (974.68-2113.16)	1313.21 (957.27-1921.76)	0.537
KLK3	872.19 (483.72-1315.05)	878.52 (478.12-1334.82)	832.76 (489.37-1252.44)	0.863
MALAT1	211.20 (67.81-1134.41)	212.22 (65.60-1110.25)	208.05 (77.89-1195.82)	0.907
PCA3	72.50 (25.80-355.68)	70.09 (24.12-369.73)	74.30 (27.60-338.45)	0.975
PSCA	5445.26 (2311.78-16043.20)	5368.99 (2186.71-15968.77)	6176.17 (2482.85-16017.93)	0.768
PSGR	49.24 (26.14-88.12)	49.91 (26.49-87.78)	47.48 (25.92-88.10)	0.717
PSMA	893.72 (607.73-1423.65)	885.46 (608.52-1412.03)	922.10 (607.94-1428.53)	0.985
USP9Y-TTTY15	12.85 (6.44-31.59)	13.13 (6.13-30.87)	12.52 (7.05-31.98)	0.565
Center — No. (%)				
Center1	308 (54.7%)	224 (56.9%)	84 (49.7%)	
Center2	152 (27.0%)	105 (26.6%)	47 (27.8%)	
Center3	103 (18.3%)	65 (16.5%)	38 (22.5%)	
Gleason Grade — No. (%)				0.993
Benign	337 (59.9%)	235 (59.6%)	102 (60.4%)	
GG 1 (GS 3 + 3)	49 (8.7%)	33 (8.4%)	16 (9.5%)	
GG 2 (GS 3 + 4)	79 (14.0%)	55 (14.0%)	24 (14.2%)	
GG 3 (GS 4 + 3)	48 (8.5%)	35 (8.9%)	13 (7.7%)	
GG 4 (all GS 8)	32 (5.7%)	23 (5.8%)	9 (5.3%)	
GG 5 (>GS 8)	18 (3.2%)	13 (3.3%)	5 (3.0%)	

* Values are median (IQR) unless indicated otherwise. IQR, interquartile range; SD, standard deviation; tPSA, total prostate-specific antigen; Center1, Tongji Hospital, Tongji Medical College, Huazhong University of Science and Technology; Center2, Changhai Hospital, Shanghai; Center3, Fujian Union Hospital, Fujian Medical University; GG, Gleason Grade; GS, Gleason score.

† The *P* values are two sided, with no adjustment for multiple comparison.

Table S6 Clinical Characteristics of the Training and Internal Validation Cohorts in tPSA 0-25 ng/mL.

Table S6. Clinical Characteristics of the Training and Internal Validation Cohorts in tPSA 0-25 ng/mL.*

Characteristic	Median (IQR)			
	**Overall (n=569)**	**Training Cohort (n=398)**	**Internal Validation Cohort (n=171)**	***P* value**†
Age — yr, mean (±SD)	66.47 ± 7.74	66.66 ± 7.94	66.03 ± 7.28	0.357
tPSA — ng/mL	9.10 (6.40-13.31)	9.15 (6.44-13.17)	9.05 (6.35-13.36)	0.968
AMACR	868.60 (429.87-1993.14)	852.34 (409.18-1990.50)	874.57 (506.82-2087.58)	0.515
DLX1	134.80 (23.20-994.47)	127.92 (22.70-1066.27)	142.40 (25.46-817.31)	0.763
FOXA1	2783.88 (2048.55-4806.54)	2747.94 (2045.33-4725.98)	2963.89 (2071.97-5112.43)	0.440
HOXB13	1339.78 (963.26-2078.83)	1347.23 (974.68-2113.16)	1306.32 (952.32-1921.59)	0.492
KLK3	872.68 (483.97-1318.59)	883.07 (478.12-1352.40)	832.76 (501.58-1234.16)	0.817
MALAT1	207.64 (67.45-1131.59)	209.90 (63.82-1110.25)	200.13 (74.35-1163.71)	0.941
PCA3	70.11 (26.28-355.27)	69.39 (24.69-369.73)	72.75 (27.68-333.93)	0.968
PSCA	5483.68 (2332.52-16017.93)	5412.06 (2206.87-15968.77)	6176.17 (2481.10-15891.84)	0.840
PSGR	49.51 (26.28-88.14)	50.59 (27.09-88.61)	47.48 (25.96-85.72)	0.611
PSMA	892.55 (607.52-1418.76)	883.01 (608.52-1391.61)	907.16 (606.26-1423.65)	0.994
USP9Y-TTTY15	13.04 (6.48-31.75)	13.25 (6.14-31.31)	12.52 (7.01-31.87)	0.672
Center — No. (%)				
Center1	309 (54.3%)	224 (56.3%)	85 (49.7%)	
Center2	154 (27.1%)	107 (26.9%)	47 (27.5%)	
Center3	106 (18.6%)	67 (16.8%)	39 (22.8%)	
Gleason Grade — No. (%)				0.983
Benign	340 (59.8%)	236 (59.3%)	104 (60.8%)	
GG 1 (GS 3 + 3)	49 (8.6%)	33 (8.3%)	16 (9.4%)	
GG 2 (GS 3 + 4)	80 (14.1%)	56 (14.1%)	24 (14.0%)	
GG 3 (GS 4 + 3)	50 (8.8%)	37 (9.3%)	13 (7.6%)	
GG 4 (all GS 8)	32 (5.6%)	23 (5.8%)	9 (5.3%)	
GG 5 (>GS 8)	18 (3.2%)	13 (3.3%)	5 (2.9%)	

* Values are median (IQR) unless indicated otherwise. IQR, interquartile range; SD, standard deviation; tPSA, total prostate-specific antigen; Center1, Tongji Hospital, Tongji Medical College, Huazhong University of Science and Technology; Center2, Changhai Hospital, Shanghai; Center3, Fujian Union Hospital, Fujian Medical University; GG, Gleason Grade; GS, Gleason score.

† The *P* values are two sided, with no adjustment for multiple comparison.

Table S7 Clinical Characteristics of the Training and Internal Validation Cohorts in tPSA 0- All ng/mL.

Table S7. Clinical Characteristics of the Training and Internal Validation Cohorts in tPSA 0- All ng/mL.*				
	Median (IQR)			
**Characteristic**	**Overall (n=586)**	**Training Cohort (n=410)**	**Internal Validation Cohort (n=176)**	***P* value**†
Age — yr, mean (±SD)	66.72 ± 7.84	66.83 ± 7.99	66.45 ± 7.50	0.581
tPSA — ng/mL	9.27 (6.45-13.58)	9.34 (6.47-13.58)	9.19 (6.39-13.54)	0.957
AMACR	877.48 (437.72-2138.92)	870.84 (411.30-2049.77)	882.60 (529.91-2269.05)	0.312
DLX1	150.23 (23.86-1052.32)	150.05 (22.70-1101.36)	155.60 (25.66-1008.36)	0.976
FOXA1	2796.92 (2051.95-4908.40)	2776.00 (2047.19-4763.61)	3008.15 (2077.91-5517.83)	0.261
HOXB13	1347.23 (969.29-2108.79)	1363.29 (980.10-2123.99)	1326.47 (964.54-1962.17)	0.696
KLK3	875.25 (483.59-1318.37)	894.12 (483.59-1345.60)	830.50 (481.99-1254.90)	0.603
MALAT1	212.22 (68.67-1218.21)	212.22 (65.60-1155.21)	219.93 (85.27-1257.42)	0.695
PCA3	75.49 (26.60-382.79)	74.43 (25.60-382.79)	75.49 (27.90-364.29)	0.889
PSCA	5648.43 (2361.26-16521.05)	5518.13 (2225.18-16478.83)	6309.03 (2514.09-16602.17)	0.631
PSGR	49.91 (26.28-89.99)	50.61 (27.78-90.56)	48.35 (25.42-87.59)	0.484
PSMA	900.74 (612.23-1464.08)	892.55 (614.47-1464.08)	924.35 (610.16-1455.40)	0.916
USP9Y-TTTY15	13.46 (6.62-32.78)	13.40 (6.14-32.38)	13.67 (7.06-36.34)	0.476
Center — No. (%).				
Center1	309 (52.7%)	225 (54.9%)	84 (47.7%)	
Center2	154 (26.3%)	107 (26.1%)	47 (26.7%)	
Center3	123 (21.0%)	78 (19.0%)	45 (25.6%)	
Gleason Grade — No. (%)				0.959
Benign	341 (58.2%)	237 (57.8%)	104 (59.1%)	
GG 1 (GS 3 + 3)	50 (8.5%)	34 (8.3%)	16 (9.1%)	
GG 2 (GS 3 + 4)	84 (14.3%)	58 (14.1%)	26 (14.8%)	
GG 3 (GS 4 + 3)	52 (8.9%)	39 (9.5%)	13 (7.4%)	
GG 4 (all GS 8)	34 (5.8%)	25 (6.1%)	9 (5.1%)	
GG 5 (>GS 8)	25 (4.3%)	17 (4.1%)	8 (4.5%)	

* Values are median (IQR) unless indicated otherwise. IQR, interquartile range; SD, standard deviation; tPSA, total prostate-specific antigen; Center1, Tongji Hospital, Tongji Medical College, Huazhong University of Science and Technology; Center2, Changhai Hospital, Shanghai; Center3, Fujian Union Hospital, Fujian Medical University; GG, Gleason Grade; GS, Gleason score.

† The *P* values are two sided, with no adjustment for multiple comparison.

Table S8 AUC (95% CI) of Models for Predicting csPCa of Urinary EVs Genes and Prostate Cancer-Associated Clinical Features in Patients of Different tPSA Levels.

Table S8. AUC (95% CI) of Models for Predicting csPCa of Urinary EVs Genes and Prostate Cancer-Associated Clinical Features in Patients of Different tPSA Levels.*										
	tPSA 0-10 ng/mL		tPSA 0-15 ng/mL		tPSA 0-20 ng/mL		tPSA 0-25 ng/mL		tPSA 0-ALL ng/mL	
Models	Training	Internal Validation	Training	Internal Validation	Training	Internal Validation	Training	Internal Validation	Training	Internal Validation
EGPS	0.938 (0.906-0.966)	0.649 (0.522-0.769)	**0.838 (0.789-0.882)**	**0.825 (0.747-0.894)**	0.838 (0.796-0.880)	0.678 (0.590-0.766)	0.846 (0.805-0.880)	0.681 (0.586-0.774)	0.831 (0.786-0.872)	0.689 (0.601-0.772)
tPSA+EGPS	0.953 (0.922-0.976)	0.564 (0.440-0.694)	0.869 (0.825-0.910)	0.798 (0.718-0.874)	0.863 (0.822-0.900)	0.719 (0.633-0.805)	0.865 (0.825-0.899)	0.703 (0.614-0.790)	0.864 (0.826-0.898)	0.714 (0.630-0.796)
CPM+EGPS	0.957 (0.927-0.982)	0.593 (0.468-0.722)	0.891 (0.853-0.926)	0.791 (0.700-0.874)	0.882 (0.845-0.917)	0.731 (0.648-0.814)	0.879 (0.840-0.912)	0.732 (0.645-0.816)	0.877 (0.842-0.910)	0.744 (0.664-0.823)
CPM	0.884 (0.831-0.927)	0.526 (0.409-0.648)	0.806 (0.761-0.851)	0.653 (0.536-0.760)	0.779 (0.730-0.824)	0.649 (0.557-0.740)	0.783 (0.733-0.828)	0.642 (0.554-0.729)	0.788 (0.737-0.832)	0.674 (0.586-0.761)
tPSA	0.852 (0.789-0.910)	0.507 (0.385-0.649)	0.792 (0.739-0.843)	0.625 (0.501-0.736)	0.773 (0.720-0.817)	0.606 (0.512-0.706)	0.775 (0.723-0.819)	0.612 (0.517-0.706)	0.784 (0.731-0.826)	0.645 (0.558-0.728)

* csPCa, clinical significantly prostate cancer; EVs, extracellular vesicles; tPSA, total prostate-specific antigen; EGPS, Extracellular vesicles Gene-based Prostate Score (including AMACR, HOXB13 and PSGR); CPM, clinical parameter model (including age and tPSA). Values in bold indicate the optimal model with the ideal AUC.

Table S9 Clinical Characteristics of the Cohorts in tPSA 0-15 ng/mL Used to Train and Validate the Model.

Table S9. Clinical Characteristics of the Cohorts in tPSA 0-15 ng/mL Used to Train and Validate the Model.*	
	**Patients with tPSA 0-15 ng/mL**
**Characteristic**	**Overall (n=483)**
Age — yr, median (IQR)	66.00 (61.00-71.00)
tPSA — ng/mL (IQR)	8.25 (6.07-11.50)
Center — No. (%)	
Center1	254 (52.6%)
Center2	135 (28.0%)
Center3	94 (19.4%)
Gleason Grade — No. (%)	
Benign	304 (62.9%)
GG 1 (GS 3 + 3)	42 (8.7%)
GG 2 (GS 3 + 4)	67 (13.9%)
GG 3 (GS 4 + 3)	37 (7.7%)
GG 4 (all GS 8)	20 (4.1%)
GG 5 (>GS 8)	13 (2.7%)

* IQR, interquartile range; tPSA, total prostate-specific antigen; Center1, Tongji Hospital, Tongji Medical College, Huazhong University of Science and Technology; Center2, Changhai Hospital, Shanghai; Center3, Fujian Union Hospital, Fujian Medical University; GG, Gleason Grade; GS, Gleason score.

Table S10 Clinical Characteristics of the Training Cohorts in tPSA 0-15 ng/mL.

Table S10. Clinical Characteristics of the Training Cohorts in tPSA 0-15 ng/mL.*			
	Median (IQR)		
Characteristic	Overall (n=338)	csPCa (n=105)	non-csPCa (n=233)
Age — yr	66.00 (61.00-71.75)	68.00 (62.00-74.00)	66.00 (60.00-70.00)
tPSA — ng/mL	8.07 (6.07-11.53)	8.74 (6.25-12.10)	8.02 (5.91-11.40)
AMACR	870.84 (427.99-1966.18)	988.97 (611.18-2059.39)	787.85 (398.32-1854.46)
DLX1	119.71 (22.00-921.66)	92.27 (17.15-872.16)	130.58 (24.10-946.22)
FOXA1	2776.00 (2047.29-4763.61)	3108.99 (2240.16-5051.06)	2718.12 (1925.15-4484.66)
HOXB13	1332.85 (953.78-2071.09)	1182.63 (901.88-1782.78)	1405.25 (973.33-2171.50)
KLK3	917.00 (483.67-1386.79)	917.00 (497.24-1443.49)	917.00 (472.64-1354.72)
MALAT1	205.07 (63.55-1110.25)	159.54 (67.34-941.15)	228.66 (63.31-1376.50)
PCA3	65.60 (25.25-292.46)	66.22 (30.83-258.25)	62.93 (23.65-338.45)
PSCA	5432.89 (2186.71-16188.39)	6383.24 (2827.59-16718.14)	5182.35 (1977.39-14211.63)
PSGR	47.46 (25.55-84.41)	57.47 (35.01-100.97)	42.18 (24.85-78.30)
PSMA	851.16 (598.20-1389.10)	908.39 (631.13-1526.26)	829.27 (570.66-1355.78)
USP9Y-TTTY15	13.60 (6.44-30.92)	18.50 (8.37-39.89)	11.80 (6.14-26.62)
Center — No. (%)			
Center1	181 (53.6%)	46 (43.8%)	135 (57.9%)
Center2	93 (27.5%)	41 (39.0%)	52 (22.3%)
Center3	64 (18.9%)	18 (17.1%)	46 (19.7%)
Gleason Grade — No. (%)			
Benign	207 (61.2%)	0 (0.0%)	207 (88.8%)
GG 1 (GS 3 + 3)	26 (7.7%)	0 (0.0%)	26 (11.2%)
GG 2 (GS 3 + 4)	52 (15.4%)	52 (49.5%)	0 (0.0%)
GG 3 (GS 4 + 3)	32 (9.5%)	32 (30.5%)	0 (0.0%)
GG 4 (all GS 8)	14 (4.1%)	14 (13.3%)	0 (0.0%)
GG 5 (>GS 8)	7 (2.1%)	7 (6.7%)	0 (0.0%)

* Values are median (IQR) unless indicated otherwise. IQR, interquartile range; csPCa clinical significantly prostate cancer; non-csPCa, non-clinical significantly prostate cancer; tPSA, total prostate-specific antigen; Center1, Tongji Hospital, Tongji Medical College, Huazhong University of Science and Technology; Center2, Changhai Hospital, Shanghai; Center3, Fujian Union Hospital, Fujian Medical University; GG, Gleason Grade; GS, Gleason score.

Table S11 Clinical Characteristics of the Internal Validation Cohorts in tPSA 0-15 ng/mL.

**Table S11. Clinical Characteristics of the Internal Validation Cohorts in tPSA 0-15 ng/mL.***			
	**Median (IQR)**		
**Characteristic**	**Overall (n=145)**	**csPCa (n=32)**	**non-csPCa (n=113)**
Age — yr	66.00 (61.00-71.00)	69.50 (61.75-72.00)	65.00 (61.00-70.00)
tPSA — ng/mL	8.63 (6.07-11.45)	10.41 (7.18-12.85)	8.17 (5.99-10.40)
AMACR	737.93 (440.21-2020.90)	988.83 (651.39-2431.49)	627.64 (411.23-1782.96)
DLX1	174.83 (26.15-1122.41)	139.55 (39.17-1026.46)	184.28 (25.86-1136.05)
FOXA1	2785.54 (2091.64-4707.63)	2923.64 (2036.97-5125.01)	2737.77 (2146.52-4628.54)
HOXB13	1307.99 (993.92-2078.83)	1243.17 (904.63-1937.51)	1398.62 (1023.20-2174.63)
KLK3	752.50 (486.66-1188.37)	694.39 (482.74-1290.81)	754.71 (486.66-1151.09)
MALAT1	216.51 (73.51-1073.26)	184.79 (62.98-641.17)	236.52 (75.15-1094.37)
PCA3	89.87 (25.56-388.91)	124.17 (59.30-552.20)	51.81 (20.94-355.27)
PSCA	5532.76 (2389.98-16068.46)	6222.13 (2457.00-24962.23)	5148.54 (2375.89-14833.11)
PSGR	47.58 (25.92-92.78)	69.26 (41.56-119.37)	43.49 (24.47-79.33)
PSMA	1010.03 (632.89-1371.51)	1039.74 (654.56-1489.75)	988.97 (610.90-1362.03)
USP9Y-TTTY15	11.79 (6.32-27.21)	13.68 (8.72-30.05)	10.04 (6.14-24.37)
Center — No. (%)			
Center1	73 (50.3%)	9 (28.1%)	64 (56.6%)
Center2	42 (29.0%)	17 (53.1%)	25 (22.1%)
Center3	30 (20.7%)	6 (18.8%)	24(21.2%)
Gleason Grade — No. (%)			
Benign	97 (66.9%)	0 (0.0%)	97 (85.8%)
GG 1 (GS 3 + 3)	16 (11.0%)	0 (0.0%)	16 (14.2%)
GG 2 (GS 3 + 4)	15 (10.3%)	15 (46.9%)	0 (0.0%)
GG 3 (GS 4 + 3)	5 (3.4%)	5 (15.6%)	0 (0.0%)
GG 4 (all GS 8)	6 (4.1%)	6 (18.8%)	0 (0.0%)
GG 5 (>GS 8)	6 (4.1%)	6 (18.8%)	0 (0.0%)

* Values are median (IQR) unless indicated otherwise. IQR, interquartile range; csPCa clinical significantly prostate cancer; non-csPCa, non-clinical significantly prostate cancer; tPSA, total prostate-specific antigen; Center1, Tongji Hospital, Tongji Medical College, Huazhong University of Science and Technology; Center2, Changhai Hospital, Shanghai; Center3, Fujian Union Hospital, Fujian Medical University; GG, Gleason Grade; GS, Gleason score.

Table S12 Clinical Characteristics of the External Validation Cohort in tPSA 0-15 ng/mL.

Table S12. Clinical Characteristics of the External Validation Cohort in tPSA 0-15 ng/mL.*			
	Median (IQR)		
Characteristic	Overall (n=59)	csPCa (n=17)	non-csPCa (n=42)
Age — yr, mean (±SD)	66.61 ± 6.78	67.71 ± 7.41	66.17 ± 6.56
tPSA — ng/mL	8.55 (6.82-10.62)	8.82 (7.54-10.80)	8.38 (6.34-10.40)
AMACR	956.28 (379.58-2067.93)	1489.04 (638.70-3214.09)	579.58 (358.24-1539.38)
DLX1	270.34 (74.61-1221.48)	665.86 (270.34-2091.71)	138.67 (44.46-1049.28)
FOXA1	4101.21 (3014.78-7330.64)	4499.93 (3018.13-13129.49)	3818.23 (3018.26-5512.12)
HOXB13	1463.65 (1166.49-1947.54)	1584.39 (1317.21-2056.79)	1316.97 (1153.15-1906.07)
KLK3	759.77 (377.28-1756.21)	707.74 (444.14-1645.63)	842.50 (362.06-1757.55)
MALAT1	304.40 (100.02-1350.73)	974.02 (148.56-1661.84)	285.66 (62.97-621.28)
PCA3	79.17 (22.19-311.14)	177.89 (72.76-331.84)	65.57 (18.40-279.00)
PSCA	9258.75 (4592.39-24063.97)	14268.64 (6148.93-62430.87)	8964.53 (4501.64-19804.09)
PSGR	57.60 (30.10-80.54)	81.81 (61.09-123.68)	41.36 (24.25-63.16)
PSMA	1368.46 (1002.72-1872.71)	1701.82 (1368.46-2015.74)	1267.43 (924.63-1704.15)
USP9Y-TTTY15	13.00 (6.82-26.37)	19.18 (7.81-33.52)	9.79 (6.06-22.57)
Center — No. (%)			
Center1	48 (81.4%)	15 (88.2%)	33 (78.6%)
Center3	11 (18.6%)	2 (11.8%)	9 (21.4%)
Gleason Grade — No. (%)			
Benign	27 (45.8%)	0 (0.0%)	27 (64.3%)
GG 1 (GS 3 + 3)	15 (25.4%)	0 (0.0%)	15 (35.7%)
GG 2 (GS 3 + 4)	8 (13.6%)	8 (47.1%)	0 (0.0%)
GG 3 (GS 4 + 3)	4 (6.8%)	4 (23.5%)	0 (0.0%)
GG 4 (all GS 8)	4 (6.8%)	4 (23.5%)	0 (0.0%)
GG 5 (>GS 8)	1 (1.7%)	1 (5.9%)	0 (0.0%)

* Values are median (IQR) unless indicated otherwise. IQR, interquartile range; SD, standard deviation; csPCa clinical significantly prostate cancer; non-csPCa, non-clinical significantly prostate cancer; tPSA, total prostate-specific antigen; Center1, Tongji Hospital, Tongji Medical College, Huazhong University of Science and Technology; Center3, Fujian Union Hospital, Fujian Medical University; GG, Gleason Grade; GS, Gleason score.

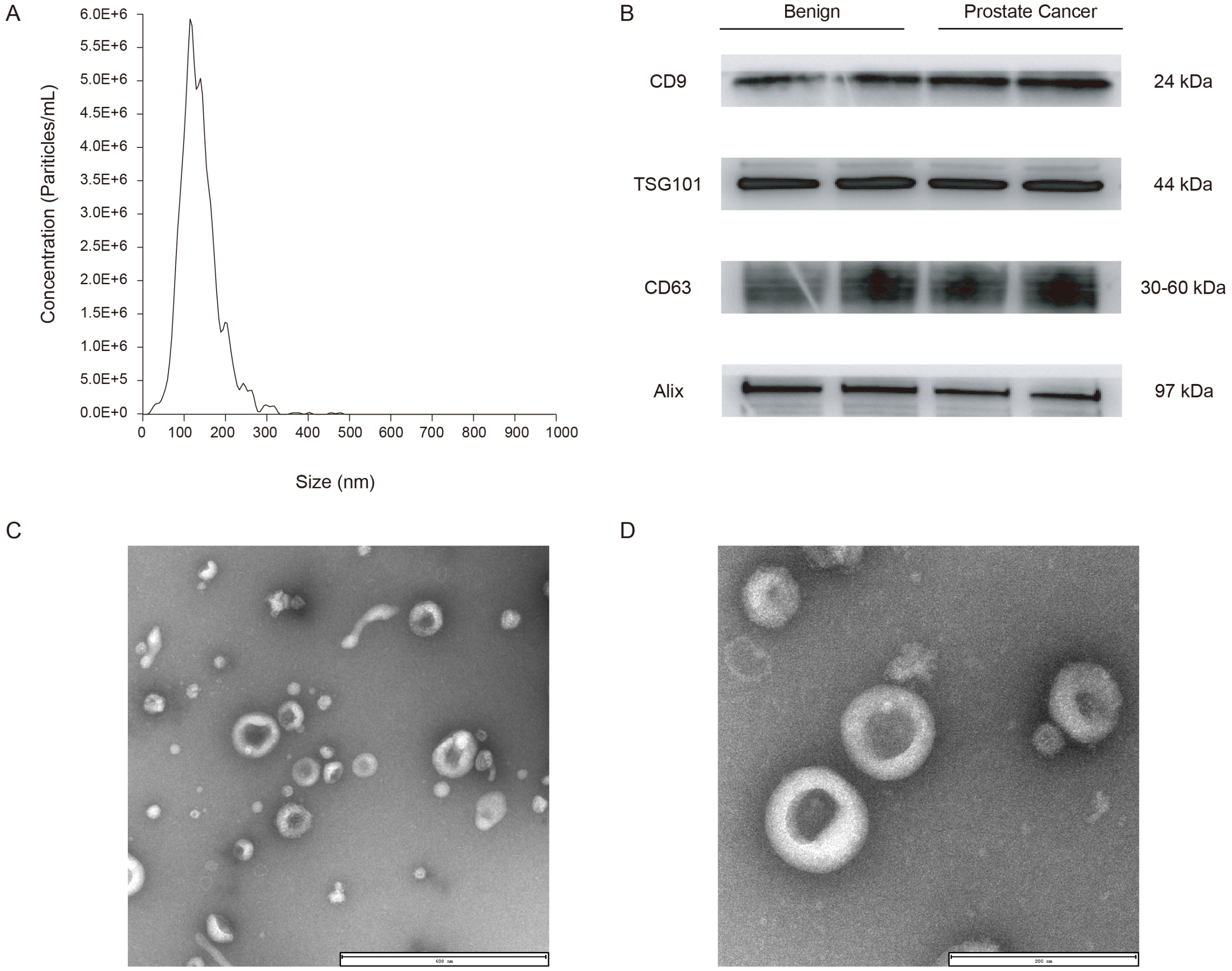

Figure S1. Characterization of EVs isolated from urine.

(A) Particle size distribution: This panel depicts the size distribution of EVs particles as determined by nanoparticle tracking (NTA) analysis. EVs particles were tested after 5000-fold dilution. (B) EVs protein analysis: Western blot (WB) analysis shows the presence of specific EVs protein markers. (C) (D) Transmission electron microscopy (TEM) morphology: Typical TEM images displaying the morphology of EVs from prostate cancer patients. (C) The scale bar is 600 nm. (D) The scale bar is 200 nm.

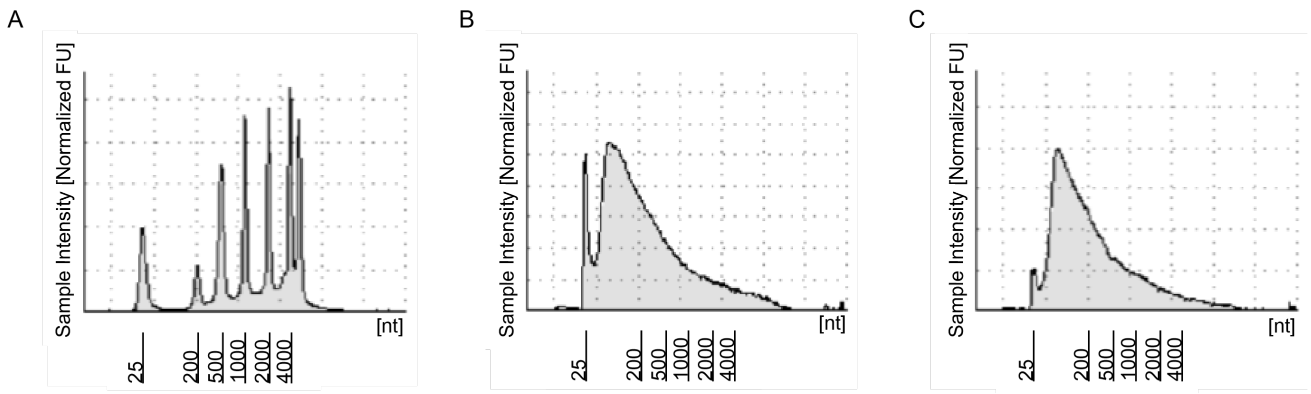

Figure S2. The electrophoresis chromatogram of total RNA analyzed by Agilent Bioanalyzer 2100.

(A) Electronic Ladder. (B) Electrophoresis chromatogram of a positive sample. The horizontal axis is the size of the nucleic acid fragment, and the vertical axis is the detection signal value. (C) Electrophoresis chromatogram of a negative sample. The horizontal axis is the size of the nucleic acid fragment, and the vertical axis is the detection signal value.

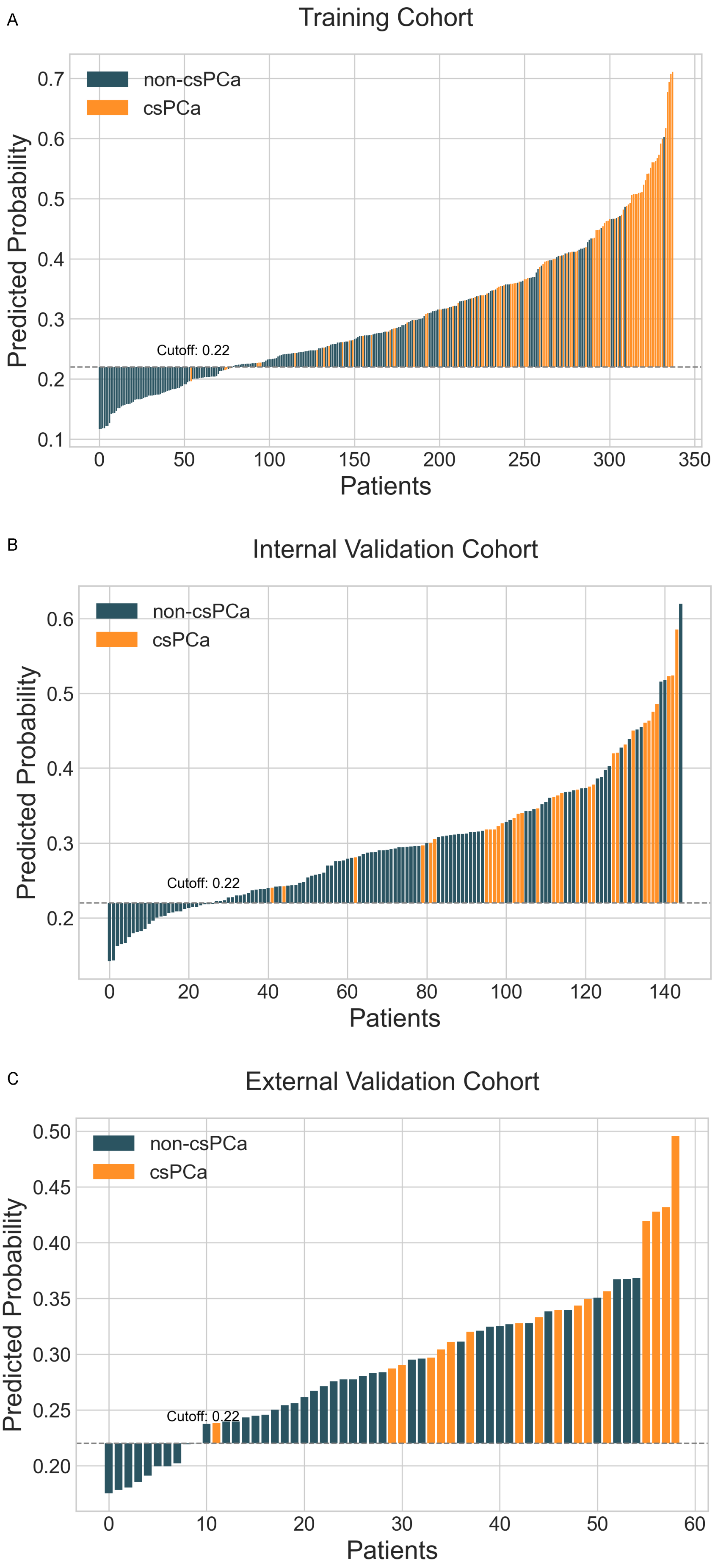
A
Training Cohort
Predicted Probability
Patients
non-csPCa
csPCa
Cutoff: 0.22
0.1
0.2
0.3
0.4
0.5
0.6
0.7
0
50
100
150
200
250
300
350
B
Internal Validation Cohort
Predicted Probability
Patients
non-csPCa
csPCa
Cutoff: 0.22
0.2
0.3
0.4
0.5
0.6
0
20
40
60
80
100
120
140
C
External Validation Cohort
Predicted Probability
Patients
non-csPCa
csPCa
Cutoff: 0.22
0.20
0.25
0.30
0.35
0.40
0.45
0.50
0
10
20
30
40
50
60

Figure S3. Waterfall plots of predicted probabilities for csPCa in different cohort using the EGPS model.

(A) Waterfall plot displaying the predicted probability of csPCa for each patient in the training cohort. Patients are sorted in ascending order based on their predicted probability. The orange bars represent true csPCa cases, while the blue bars indicate non-csPCa cases. The cut-off value for classification is set at 0.22. (B) Waterfall plot showing the predicted probability of csPCa in the internal validation cohort. (C) Waterfall plot showing the predicted probability of csPCa in the external validation cohort. Abbreviations: csPCa, clinical significantly prostate cancer.

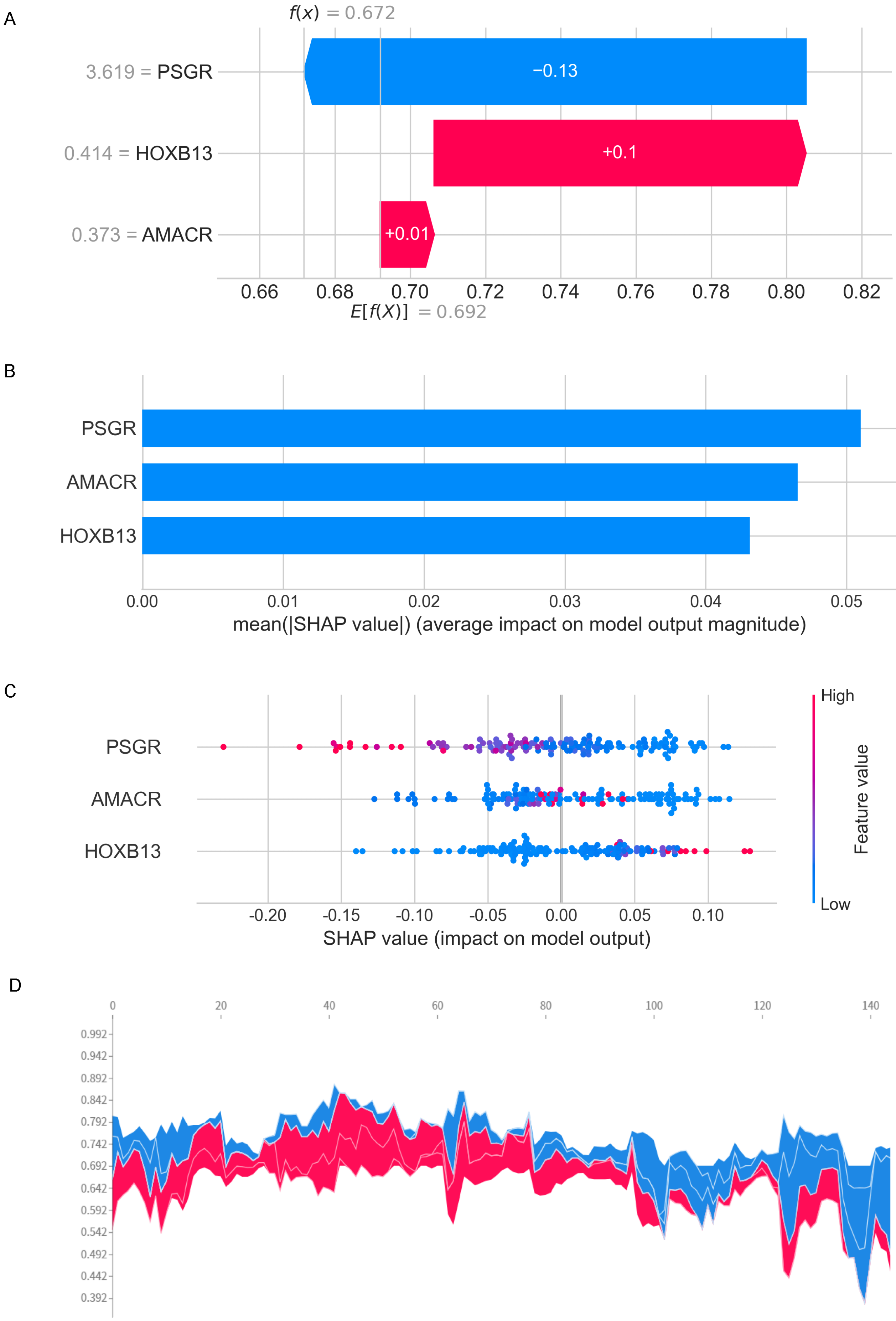
A
f(x) = 0.672
3.619 = PSGR
0.414 = HOXB13
0.373 = AMACR
−0.13
+0.1
+0.01
0.66
0.68
0.70
0.72
0.74
0.76
0.78
0.80
0.82
E[f(X)] = 0.692
B
PSGR
AMACR
HOXB13
0.00
0.01
0.02
0.03
0.04
0.05
mean(|SHAP value|) (average impact on model output magnitude)
C
PSGR
AMACR
HOXB13
-0.20
-0.15
-0.10
-0.05
0.00
0.05
0.10
SHAP value (impact on model output)
High
Feature value
Low
D
0
20
40
60
80
100
120
140
0.992
0.942
0.892
0.842
0.792
0.742
0.692
0.642
0.592
0.542
0.492
0.442
0.392

Figure S4. XAI analysis of model in predicting csPCa of tPSA 0-15 ng/mL.

Feature importance analysis using SHAP values provided insights into the model's decision-making process. The explainability analysis was conducted on the EGPS model within the tPSA 0-15 ng/mL subgroup to evaluate the contributions of individual features to the prediction outcome (A) For an individual patient, SHAP analysis illustrated how different features contributed to the final prediction score. A positive SHAP value indicated a feature positively influencing the prediction towards positive outcome, while a negative value indicated an opposing effect. In the case shown in Figure S4A, AMACR had the highest negative contribution, whereas HOXB13 and PSGR exhibited relatively weaker positive effects on the model's prediction. (B) Across the entire dataset, Random Forest-based SHAP value analysis ranked PSGR as the most influential feature for csPCa prediction, followed by AMACR and HOXB13. The mean absolute SHAP values provided a measure of each feature's overall impact on model predictions. (C) The distribution diagram of SHAP values demonstrated how each feature influenced individual predictions. Higher feature values were denoted in red, while lower values were in blue. A positive SHAP value indicated a feature's positive contribution to the csPCa prediction, whereas negative SHAP values suggested a suppressive effect. As mentioned above, the results suggest that higher PSGR expression is more likely to lead the model to predict a negative result for csPCa. (D) To further illustrate the cumulative effect of feature importance across multiple patients, a force plot was generated, showing how each feature influenced the final predictions dynamically. The variation in SHAP values across different patients highlighted the heterogeneity of feature contributions in csPCa prediction.
